# Prognostic impact of stage reclassification and N2 subclassification in the ninth edition of TNM classification for non-metastatic lung cancer

**DOI:** 10.1007/s12149-026-02198-w

**Published:** 2026-03-28

**Authors:** Muhammet Halil Baltacioglu, Demet Nak, Pelin Sahin Oguz, Ogün Bulbul

**Affiliations:** 1 Department of Nuclear Medicine, Trabzon Kanuni̇ Trai̇ni̇ng and Research Hospi̇tal, Trabzon, Turkey; 2 Department of Nuclear Medicine, Recep Tayyip Erdoğan University Training and Research Hospital, Rize, Turkey

**Keywords:** TNM 9 staging, Lung cancer, ^18^F-FDG PET/CT, Prognosis

## Abstract

**Purpose:**

This study aimed to evaluate whether the 9th edition of the tumor–node–metastasis (TNM) staging system more accurately reflects prognosis than the 8th edition in patients with non-metastatic lung cancer staged clinically using ^18^F-fluorodeoxyglucose positron emission tomography/computed tomography (^18^F-FDG PET/CT).

**Material-methods:**

In this retrospective, two-center study, 1,590 patients diagnosed with lung cancer were initially evaluated. After excluding those with distant metastases at diagnosis, 529 patients (mean age: 65.9 ± 8.7 years) with stage I–III non-metastatic disease who underwent ^18^F-FDG PET/CT between February 2017 and December 2023 were included. All patients were reclassified according to both the 8th and 9th editions of the TNM system, and changes in staging (upstaging, downstaging, or unchanged) were analyzed in relation to overall survival (OS).

**Results:**

The 5-year OS rates were 75.3% for cN0, 55.9% for cN1, 50.0% for cN2a, and 33.7% for cN2b. Although cN2a patients had higher OS compared to cN2b, the difference was not statistically significant (*p* = 0.20). Importantly, patients with T1N2a tumors who were down-staged from stage IIIA to IIB showed significantly lower OS compared to those consistently classified as stage IIB (*p* = 0.029), suggesting prognostic mismatch. In contrast, survival trends for patients down-staged from IIB to IIA (T1N1) and up-staged from IIIA to IIIB (T2N2b) were consistent with the revised TNM 9 classification, though without statistical significance (*p* = 0.19 and *p* = 0.085, respectively).

**Conclusion:**

While several staging changes in the 9th TNM edition align with prognostic expectations, the downstaging of the T1N2a subgroup appears to be inconsistent with actual survival outcomes. These findings highlight the need for further large-scale, prospective, multicenter studies to validate the clinical reliability and implications of the revised staging system.

## Introduction

Lung cancer remains the highest cause of cancer-related deaths worldwide, affecting millions of people each year. According to data for 2022 alone, approximately 2.5 million new cases and more than 1.8 million deaths have been reported, demonstrating the global significance of this disease [1]. Therefore, comprehensive and accurate staging is of great importance to effectively treat lung cancer and accurately predict patient outcomes.

**Fig. 1:**
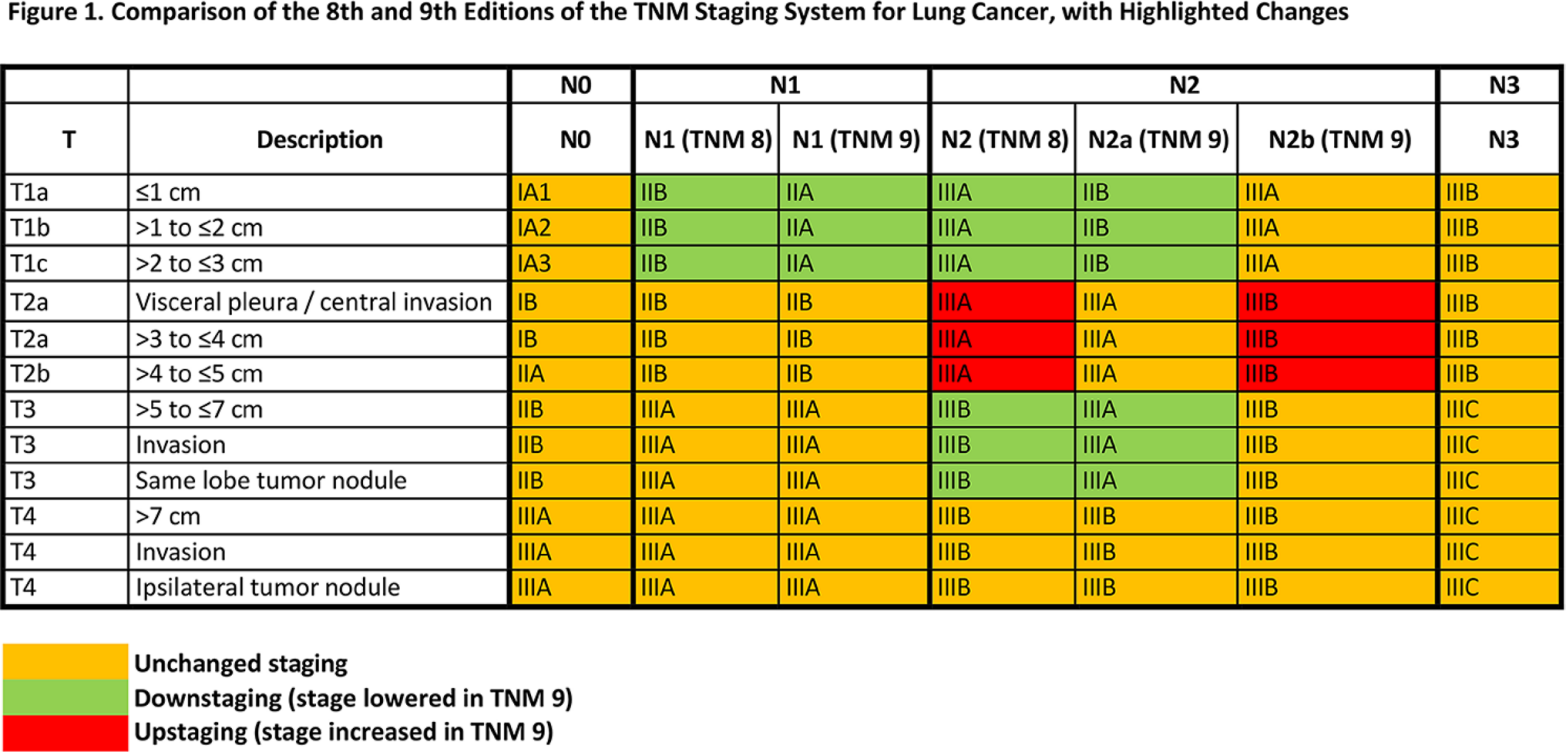
Comparison of the 8th and 9th Editions of the TNM Staging System for Lung Cancer, with Highlighted Changes. This table illustrates the modifications introduced in the 9th edition, including the subdivision of the N2 category into N2a (single station) and N2b (multiple stations). Orange cells indicate unchanged staging, green cells represent downstaging (stage lowered in TNM 9), and red cells highlight upstaging (stage increased in TNM 9). Notably, T1N1 has been downstaged from stage IIB to IIA, T1N2a from stage IIIA to IIB, and T3N2a from stage IIIB to IIIA, reflecting a reclassification that places these tumors in lower prognostic categories. Conversely, T2N2b has been upstaged from stage IIIA to IIIB

The tumor-node-metastasis (TNM) classification system was first introduced by the American Joint Committee on Cancer (AJCC) and Union for International Cancer Control (UICC) in 1987 and constitutes the standard framework for the classification of lung cancer according to its anatomical spread [2]. Over the years, continuous revisions have been applied, especially in the definition of ‘T’, to reflect the prognosis of lung cancer patients without metastasis more accurately. However, the definition of ‘N’, which defines lymph node involvement, has not significantly changed for a long time. Since the introduction of the N3 category in 1987, nodal staging has been based mainly on the anatomical location of the affected lymph nodes and has not included additional details [3, 4]. This limits the reliability of the staging system by causing patients in the same N stage to have different results. To overcome this problem, the ninth edition of the TNM classification, which officially came into effect in 2024 according to UICC/AJCC and has begun to be adopted in routine clinical practice, introduced an important change by subdividing the N2 category into single-station (N2a) and multiple-station (N2b) involvement [5]. This change will allow more accurate differentiation of patient outcomes, contributing to improved treatment decisions and a clearer understanding of prognosis [6, 7]. For example, tumors previously classified as T1N1M0, T1N2aM0, and T3N2aM0 were downstaged to stages IIA, IIB, and IIIA, respectively, while tumors classified as T2N2bM0 were upstaged from stage IIIA to stage IIIB. These modifications may have important implications on patient management and prognostic assessment, especially for stage II and III patients [8]. However, additional research is needed to confirm how these changes, especially stage transitions, will affect survival outcomes beyond the current data presented by the International Association for the Study of Lung Cancer (IASLC). A summary of staging changes between the eighth and ninth editions is presented in Fig. 1.

The aim of this study was to evaluate whether the revisions introduced in the 9th edition of the TNM staging system better reflect prognosis compared to the 8th edition in patients with non-metastatic lung cancer staged clinically using ^18^F-fluorodeoxyglucose positron emission tomography/computed tomography (^18^F-FDG PET/CT).

## Materials and methods

### Patient population

This retrospective, two-center study analyzed imaging data from 1,590 patients diagnosed with lung cancer. All patients underwent ^18^F-FDG PET/CT, brain MRI and thoracic CT, abdominal CT, or MRI for staging purposes. Patients showing distant metastasis at the initial diagnosis were excluded from the study, resulting in a final cohort of 529 patients (mean age: 65.9 ± 8.7 years) who were diagnosed with stage I–III lung cancer and underwent staging with ^18^F-FDG PET/CT between February 2017 and December 2023. All patients were staged using both the eighth and ninth editions of the TNM staging system. The staging classifications of patients whose stage changed under the new system were compared both with their previous TNM 8 stage counterparts and with other patients classified within the same TNM 9 stage. Nodal staging in this study was based on clinical assessment derived from ^18^F-FDG PET/CT. Pathological nodal information was not uniformly available for all patients and was therefore not used for primary stage assignment in this PET/CT-based cohort. Treatment data were retrieved from institutional medical records and categorized as surgery, chemotherapy, radiotherapy, or combined modality therapy. Ethical approval for this study was obtained from the Scientific Research Ethics Committee of the University of Health Sciences, Trabzon Faculty of Medicine (Meeting No: 2025/04; Decision No: 10496660-209).

### ^18^F-FDG PET/CT imaging

All PET/CT imaging studies were performed using a GE Discovery 710 PET/CT scanner (GE Medical Systems, Milwaukee, USA) and Biograph 20 mCT PET/CT scanner (Siemens Healthcare, Germany), following standard guidelines for tumor imaging [9]. Patients fasted for at least 6 h before imaging, and blood glucose levels were measured prior to radiotracer injection. Patients received an intravenous injection of 5.5 MBq/kg ^18^F-FDG and rested for approximately 60 min in a quiet, lead-lined room with semi-recumbent chairs to minimize background activity and radiation exposure. PET images were acquired at 4 min per bed position. A low-dose CT scan was performed prior to PET imaging using the following parameters: tube voltage 140 kV, tube current 70 mA, rotation time 0.5 s per rotation, pitch of 6, and slice thickness of 5 mm.

### Image and data analysis and statistical analyses

Reconstructed PET/CT images were retrospectively reviewed on a dedicated workstation (Advance Workstation 4.7, GE Medical Systems, Milwaukee, USA), including maximum intensity projection (MIP), PET, CT, and fused PET/CT images in axial, coronal, and sagittal planes. ^18^F-FDG uptake was assessed in both primary lung tumors and lymph node metastases, and patients were staged according to the 8th and 9th TNM editions. Mediastinal and hilar lymph nodes were assessed on ^18^F-FDG PET/CT using a combined visual and semiquantitative approach. Lymph nodes were considered suspicious when ^18^F-FDG uptake exceeded mediastinal blood pool activity and was discordant with normal physiologic distribution, in conjunction with morphologic features on the low-dose, non-contrast CT component such as short-axis diameter ≥ 10 mm or rounded configuration. Nodes with imaging and characteristics suggestive of infectious or granulomatous disease were not considered malignant and were excluded from staging analyses. No fixed SUVmax cutoff was applied; rather, interpretation relied on integrated clinical assessment. All PET/CT examinations were independently reviewed by two board-certified nuclear medicine physicians who were blinded to each other’s interpretations. In cases of disagreement between the reviewers, the final decision was made by consensus among the specialists. Overall survival (OS) was calculated from the date of the initial ^18^F-FDG PET/CT examination until the date of death or the end of the follow-up period for surviving patients.

Baseline clinical and demographic characteristics were evaluated using descriptive statistics. Categorical variables were presented as frequencies and analyzed using the chi-square test. Continuous variables were reported as mean ± standard deviation (SD) or median with interquartile range (IQR). The normality of data distribution was assessed using the Kolmogorov–Smirnov test. Kaplan-Meier analysis was used to calculate overall survival (OS), and the log-rank test was performed for comparisons between groups. Statistical significance was defined as a p-value ≤ 0.05. All statistical analyses were performed using SPSS version 28.0 software.

### Findings

Five hundred twenty-nine patients (mean age of 65.9 ± 8.7 years) participated in this study. Most of the patients were male (*n* = 474, 90%). Demographic, histopathological, staging characteristics, and overall treatment distributions are summarized in Table 1.

**Table 1:** Demographic data of study group

	**(n = 529)**
Age, mean + SD (years)	65.9 ± 8.7
Gender, n (%)	
Male	474 (90)
Female	55 (10)
Histopathology, n (%)	
Adenocarcinoma	181 (34)
Squamous cell carcinoma	236 (45)
Small cell carcinoma	54 (10)
Others	58 (11)
Treatment modalities, n (%)	
Chemotherapy	373 (74)
Radiotherapy	294 (58)
Surgery	210 (42)
No treatment	16 (3)
Missing	25 (5)
8th TNM staging system, n (%)	
I	104 (19)
IIA	25 (5)
IIB	67 (13)
IIIA	119 (23)
IIIB	134 (25)
IIIC	80 (15)
9th TNM staging system, n (%)	
I	104 (19)
IIA	34 (7)
IIB	65 (12)
IIIA	112 (22)
IIIB	134 (25)
IIIC	80 (15)
SD: Standard Deviation	

Treatment information was available for 504 patients (95.3%), while data were unavailable for 25 patients (4.7%). Among patients with available treatment data, 373 (74.0%) received chemotherapy, 294 (58.3%) radiotherapy, and 210 (41.7%) underwent surgical resection. With respect to treatment strategies, 46 patients (8.7%) received chemotherapy alone, 17 (3.2%) radiotherapy alone, and 88 (16.6%) surgery alone. Combined chemoradiotherapy was the most frequent approach (*n* = 215, 40.6%). Chemotherapy plus surgery was administered in 60 patients (11.3%), surgery plus radiotherapy in 10 (1.9%), and trimodality therapy (chemotherapy, radiotherapy, and surgery) in 52 patients (9.8%). Sixteen patients (3.0%) did not receive active oncologic treatment, mainly due to poor performance status, comorbidities, advanced age, or patient preference.

On clinical staging based primarily on ^18^F-FDG PET/CT and cross-sectional imaging, 39% (*n* = 205) of patients were classified as cN0 disease, while 12% (*n* = 63), 28% (*n* = 149), and 21% (*n* = 112) had cN1, cN2, and cN3 disease, respectively. Of the patients diagnosed with cN2 disease, according to the ninth edition of the TNM classification, 44% (*n* = 65) were categorized as cN2a and 56% (*n* = 84) as cN2b. A comparison of patient numbers and stage migrations according to the eighth and ninth editions of the TNM staging system is shown in Table 2.

**Table 2:**
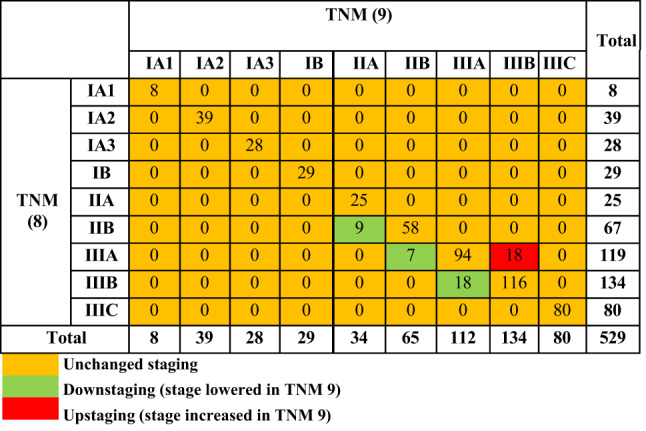
Comparison of Patient Numbers and Stage Changes According to the 8th and 9th Editions of the TNM Staging System

During the follow-up period, 305 patients (58%) died from lung cancer. The median duration of follow-up was 41.2 months (range: 2.2–224 months).

**Fig. 2:**
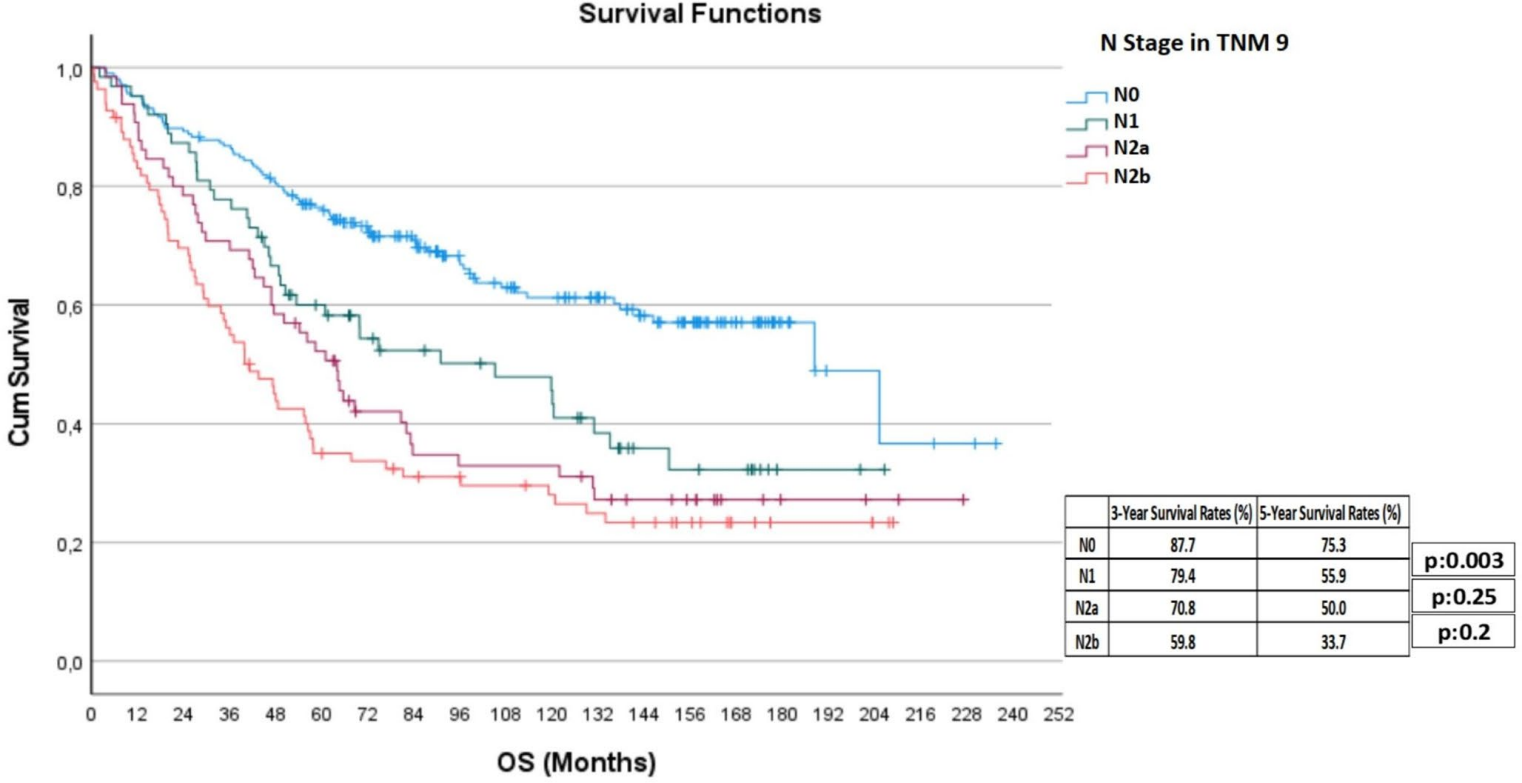
Overall survival (OS) according to N stage in TNM 9 staging. Kaplan-Meier curves show survival in N0, N1, N2a, and N2b stages. Survival significantly decreases with increasing stage. Survival is highest in the N0 stage and lowest in the N2b stage. The table shows 3 and 5-year survival rates for each N stage. The difference between N0 and N1 is statistically significant (*p* = 0.003), whereas the comparisons between N1 and N2a (*p* = 0.25) and between N2a and N2b (*p* = 0.20) are not significant

Kaplan–Meier survival analysis demonstrated that the 3-year OS rates for cN0, cN1, cN2a, and cN2b patients were 87.7%, 79.4%, 70.8%, and 59.8%, respectively, while the corresponding 5-year OS rates were 75.3%, 55.9%, 50.0%, and 33.7%, respectively. Although patients with cN2a had better OS than cN2b, the difference was not statistically significant (*p* = 0.20). In addition, the difference in OS between cN0 and cN1 was statistically significant (*p* = 0.003) (Fig. 2). When stratified by histology, the 5-year OS rates for cN0, cN1, cN2a, and cN2b were 78.8%, 65.2%, 42.5%, and 34.4% in patients with squamous cell carcinoma (SCC; *n* = 236), and 78.0%, 63.4%, 55.0%, and 47.8% in those with adenocarcinoma (*n* = 181), respectively, indicating that absolute survival differed by histologic subtype while the stepwise decline across nodal subcategories was preserved. Within histologic subgroups, the survival difference between cN2a and cN2b did not reach statistical significance in either SCC (*p* = 0.50) or adenocarcinoma (*p* = 0.51). Other histologic subtypes were not analyzed separately because of small subgroup sizes, which limited statistical power for meaningful comparison.

**Fig. 3:**
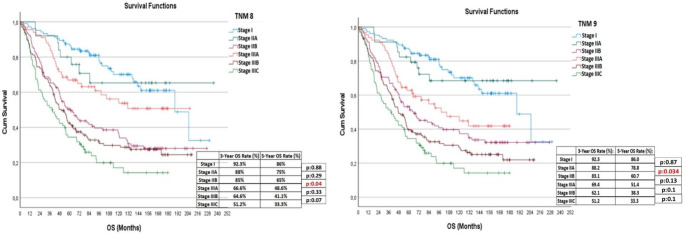
Comparison of TNM 8 and TNM 9 staging systems regarding overall survival. The graph shows that survival decreases as the stage increases in both staging systems. The tables show 3- and 5-year survival rates for each stage and p-values ​​for comparisons. There was a statistically significant difference in OS between stage IIB and IIIA in TNM 8 and between stage IIA and IIB in TNM 9, with p-values of 0.04 and 0.034, respectively

We evaluated the prognostic impact of the revised TNM staging system by comparing the 5-year OS rates between the eighth and ninth editions (Fig. 3). The survival rates demonstrated a progressive decline with advancing stages in both editions. Most notably, the differences between adjacent stage groups reached statistical significance between stage IIB and stage IIIA in TNM 8 (*p* = 0.04) and between stage IIA and stage IIB in TNM 9 (*p* = 0.034).

**Fig. 4:**
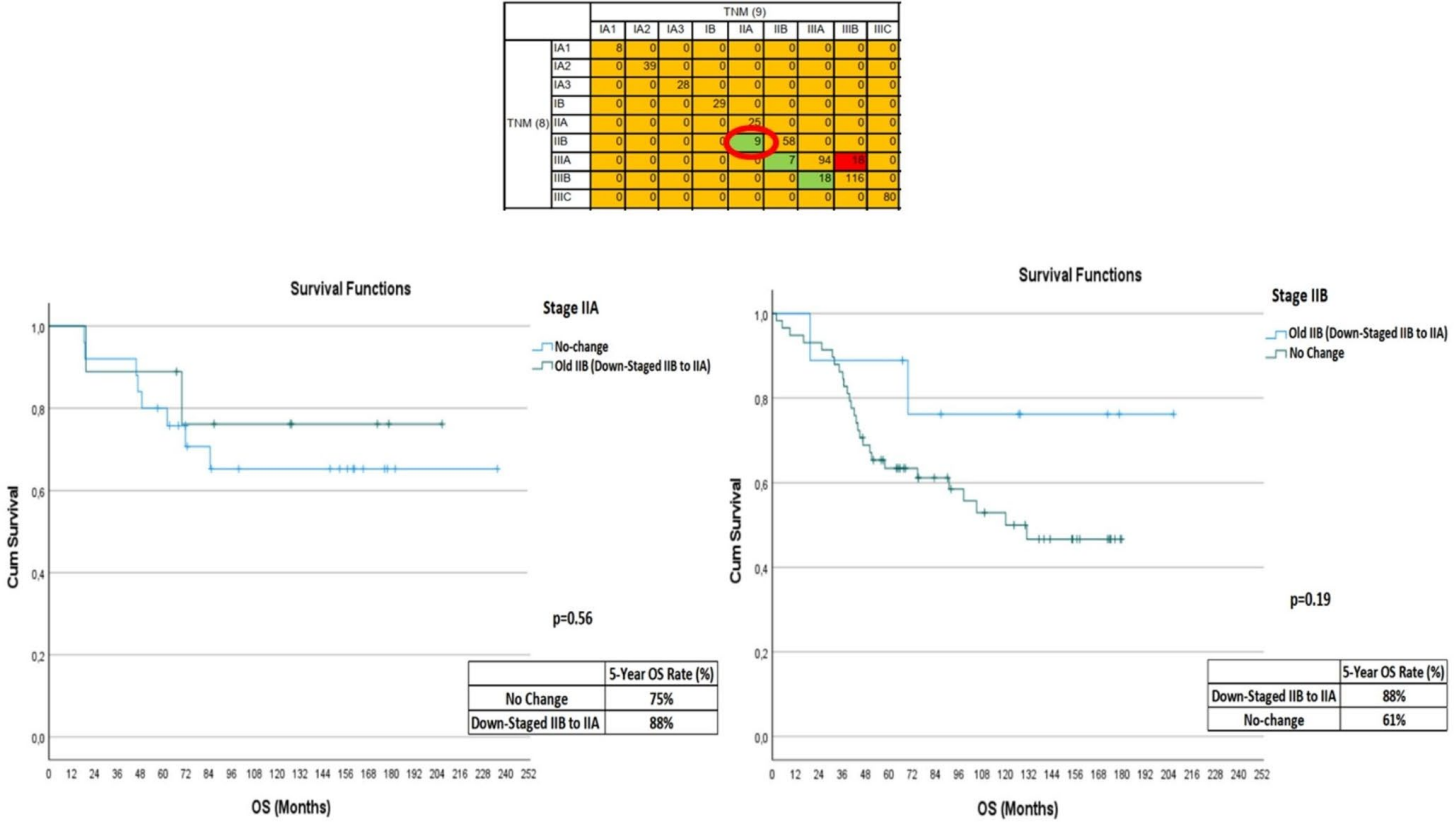
Survival Impact of Down-staging from IIB to IIA in TNM 9. The top panel shows the distribution of stage migration from TNM 8 to TNM 9, highlighting cases that were down-staged or up-staged. The bottom panel presents Kaplan-Meier survival curves comparing OS between patients whose stage remained unchanged in IIA and those who were down-staged from IIB to IIA. Stage IIA (left graph): Down-staged patients had a higher OS compared to patients whose stage remained unchanged in IIA, but this was not statistically significant (*p* = 0.56). The 5-year OS was 88% down-staged group and 75% was an unchanged group. Stage IIB (right graph): Down-staged patients also had better survival, but again, the difference was not statistically significant (*p* = 0.19). The 5-year OS was 88% down-staged group and 61% was unchanged group

When analyzing the stage migrations individually, the 5-year OS rate for patients consistently staged as IIA (TNM 8–9) was 75.0%. In comparison, the OS rate for those down-staged from IIB to IIA increased to 88.0%. Survival outcomes of the down-staged group were comparable to those of consistent IIA patients (*p* = 0.56). Although their OS rate was higher than patients consistently staged as IIB (TNM 8–9), the difference was not statistically significant (*p* = 0.19) (Fig. 4).

**Fig. 5:**
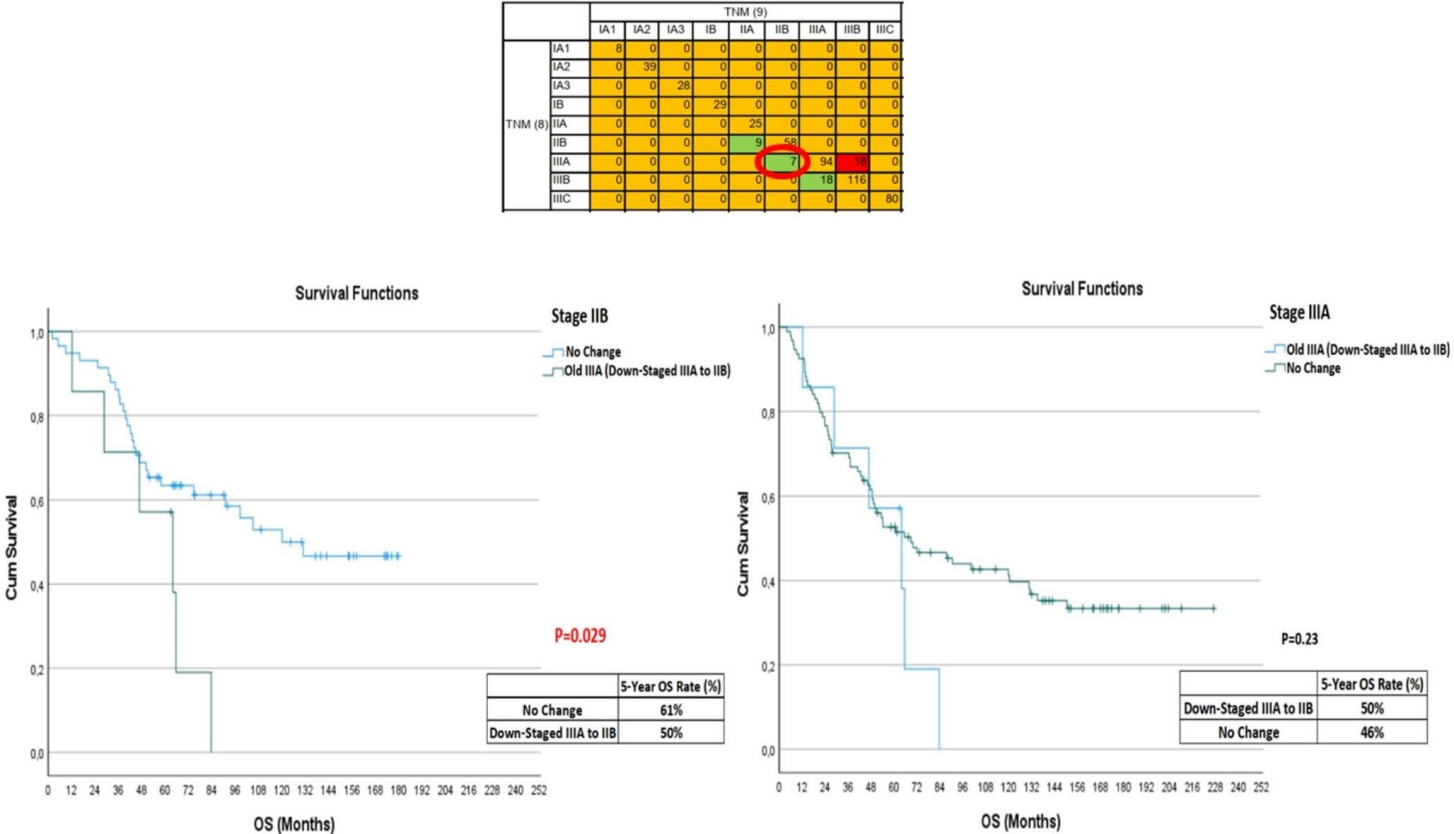
Survival Impact of Downstaging from IIIA to IIB in TNM 9. The top panel displays stage migration between TNM 8 and TNM 9, highlighting cases that were down-staged from IIIA to IIB. The bottom panel presents Kaplan-Meier survival curves comparing OS for patients whose stage remained unchanged in IIB versus those down-staged from IIIA to IIB. Stage IIB (left graph): Patients down-staged from IIIA to IIB had a lower OS compared to patients whose stage remained unchanged in IIB, and this difference was statistically significant (*p* = 0.029). The 5-year OS was 50% down-staged group and 61% was unchanged group. Stage IIIA (right graph): Survival between down-staged and an unchanged cases was similar with no significant difference (*p* = 0.23). The 5-year OS was 50% down-staged group and 46% was unchanged group

For patients staged as IIB consistently (TNM 8–9), the 5-year OS rate was 61.0%, while patients down-staged from IIIA to IIB had a lower OS rate of 50.0%, which was similar to the OS rate of patients consistently staged as IIIA (46.0%). The OS of down-staged IIIA to IIB (T1N2aM0) was significantly lower than that of TNM 8–9 IIB (*p* = 0.029), whereas it was similar to that of TNM 8–9 IIIA (*p* = 0.23) (Fig. 5).

**Fig. 6:**
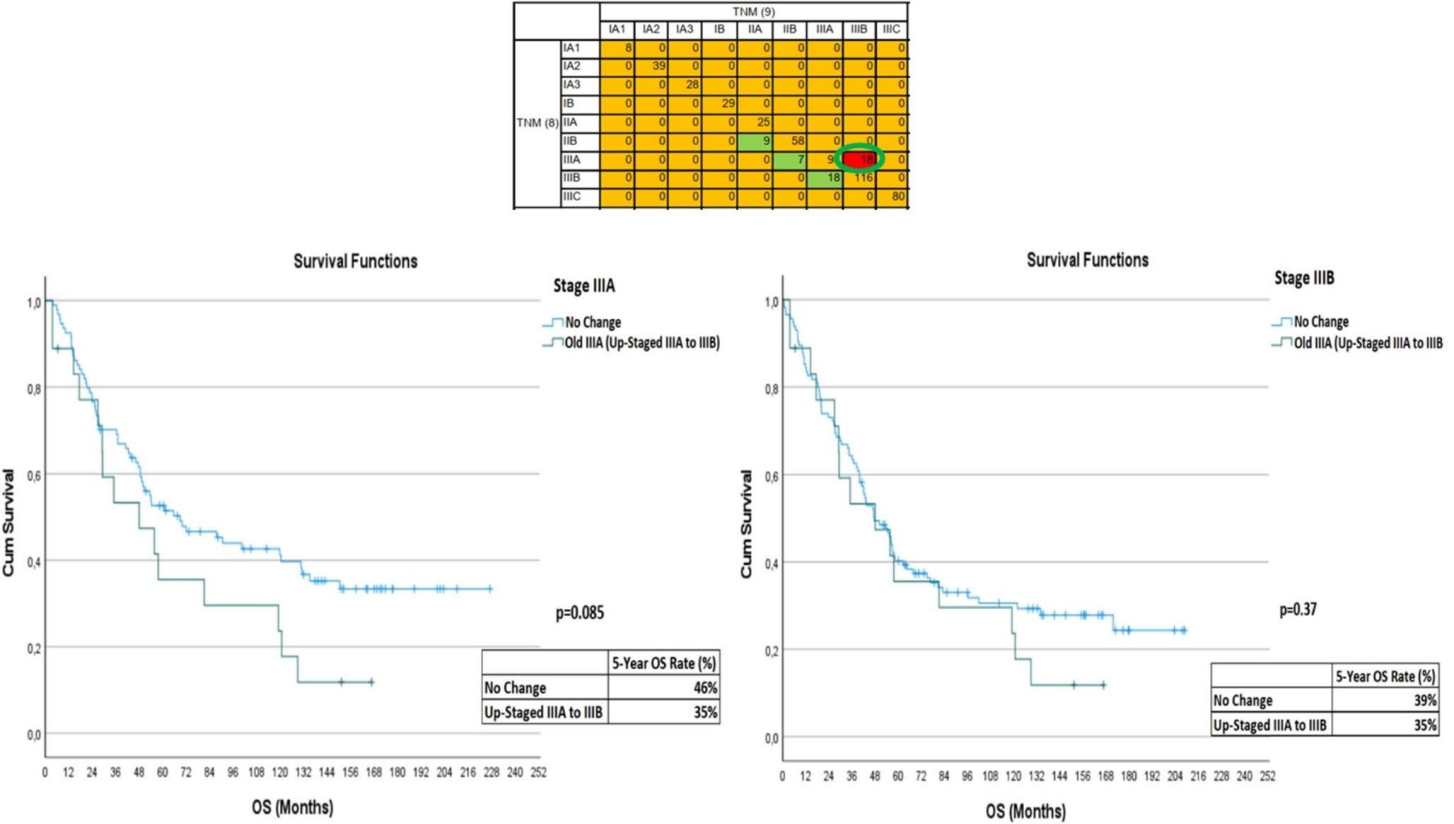
The top panel illustrates stage migration from TNM 8 to TNM 9, highlighting cases upstaged from IIIA to IIIB. The bottom panel presents Kaplan-Meier survival curves comparing OS between patients whose stage remained unchanged in IIIA or IIIB and those up-staged from IIIA to IIIB. Stage IIIA (left graph): Upstaged patients had a lower OS compared to patients whose stage remained unchanged in IIIA, but the difference was not statistically significant (*p* = 0.085). The 5-year OS was 35% up-staged group and 46% was an unchanged group. Stage IIIB (right graph): OS was similar between the up-staged group and patients whose stage remained unchanged (*p* = 0.37). 5-year OS was 35% up-staged group and 39% was an unchanged group

The 5-year OS rates were 46.0% for TNM 8–9 IIIA, 35.0% for patients up-staged from IIIA to IIIB (T2N2bM0), and 39.0% for TNM 8–9 IIIB. Although the OS of up-staged IIIA to IIIB was lower than that of TNM 8–9 IIIA, this difference was not statistically significant (*p* = 0.085). Additionally, the 5-year OS of up-staged IIIA to IIIB was similar to that of TNM 8–9 IIIB, without statistical significance (*p* = 0.37) (Fig. 6).

**Fig. 7:**
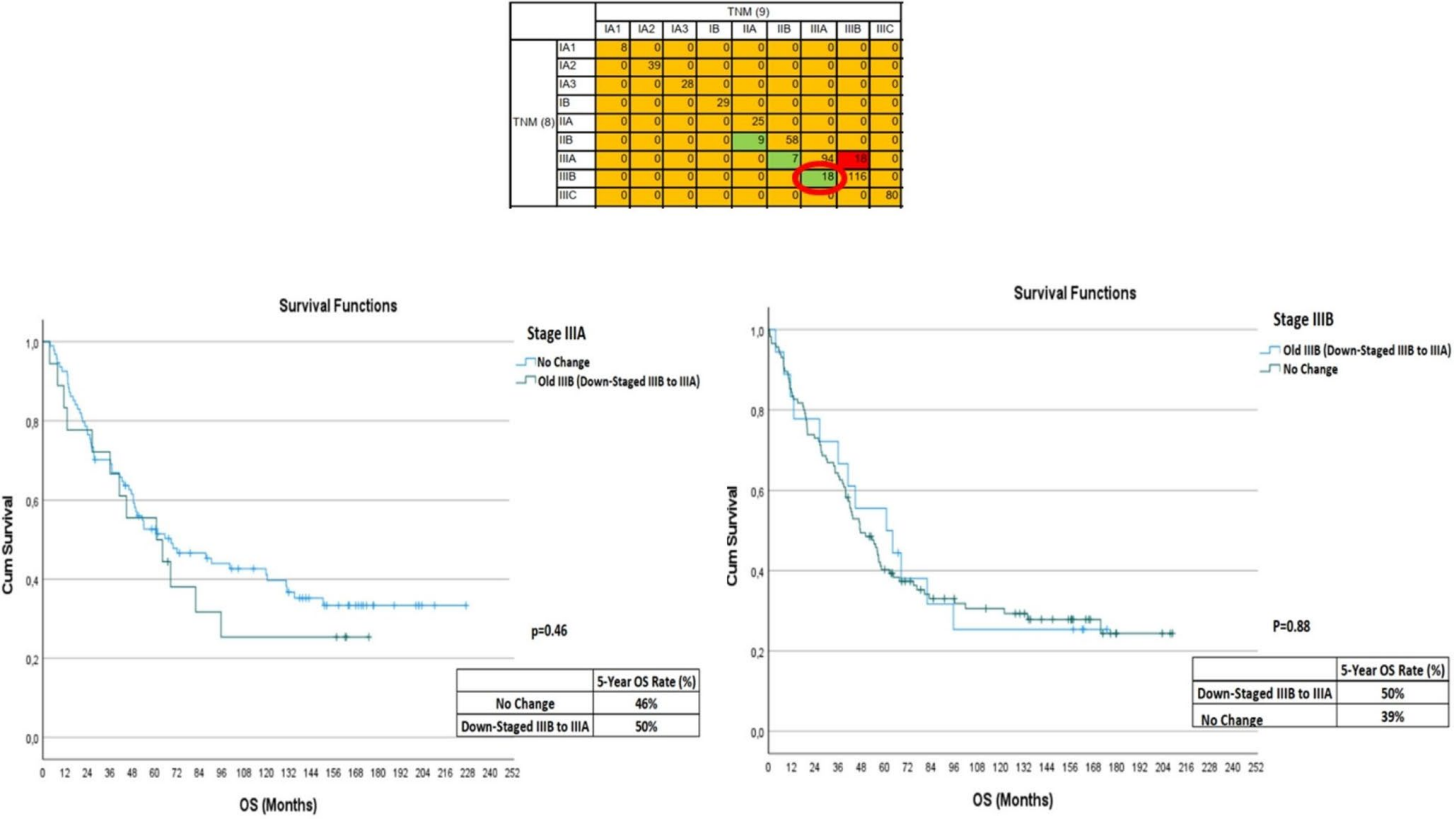
Survival Impact of Downstaging from IIIB to IIIA in TNM 9. The top panel displays stage migration from TNM 8 to TNM 9, highlighting cases down-staged from IIIB to IIIA. The bottom panel presents Kaplan-Meier survival curves comparing OS between patients whose stage remained unchanged in IIIA or IIIB and those down-staged from IIIB to IIIA. Stage IIIA (left graph): There was no statistically significant difference in overall survival between the down-staged group and the group whose stage remained unchanged (*p* = 0.46). The 5-year OS was 50% down-staged group and 46% was an unchanged group. Stage IIIB (right graph): Survival between down-staged and unchanged cases was similar with no significant difference (*p* = 0.88). The 5-year OS was 50% down-staged group and 39% was an unchanged group

Lastly, there was no significant difference in OS among patients who were down-staged from stage IIIB to IIIA (T3N2aM0), both TNM 8–9 IIIA and IIIb (respectively *p* = 0.46, 0.88). However, the 5-year OS rates were 46% for stage IIIA, 50% for patients downstaged from IIIB to IIIA, and 39% for stage IIIB (Fig. 7).

## Discussion

Accurate staging in lung cancer is crucial for prognosis prediction and treatment decision-making. In the 9th edition of the TNM classification, the N2 stage was subdivided into N2a (single station) and N2b (multiple stations) in order to improve prognosis assessment in N2 disease. This change has led to stage transitions in specific tumor-node configurations, both up-staging and down-staging. In this study, we analyzed the impact of these changes on OS in a cohort of 529 patients with non-metastatic lung cancer to determine whether the new classification accurately reflects survival differences.

In our cohort, nodal staging was performed primarily by ^18^F-FDG PET/CT–based clinical assessment. Patients with cN2a disease demonstrated numerically better OS than those with cN2b disease; however, this difference did not reach statistical significance (*p* = 0.20), with 5-year OS rates of 50.0% and 33.7%, respectively. These findings suggest a directionally consistent but attenuated prognostic separation within a clinically staged population. By comparison, the IASLC ninth-edition staging analyses reported by Huang et al. demonstrated a statistically robust distinction between N2a and N2b among clinically staged patients, with 5-year OS of approximately 42% for cN2a and 31% for cN2b and a hazard ratio of approximately 1.27 favoring single-station disease (*p* < 0.001) [5]. Thus, while the adverse prognostic impact of multistation involvement is concordant with our results, the IASLC cohort showed stronger discrimination despite lower absolute survival for cN2a than in our series. Notably, clinical nodal staging in the IASLC dataset was not mandated to rely specifically on ^18^F-FDG PET/CT, which may further contribute to differences in staging performance across cohorts. Differences were even more pronounced in surgically staged populations. In IASLC pathological subsets, Huang et al. reported 5-year OS of approximately 51% for pN2a and 40% for pN2b, with a hazard ratio of approximately 1.46 (*p* < 0.001) [5]. Additional validation cohorts further illustrate the heterogeneity of prognostic separation between N2a and N2b. In an external validation cohort reported by Son et al., the 5-year OS rates were 63.6% for pN2a and 53.1% for pN2b (*p* = 0.128) [10]. Similarly, Nakao et al. observed no statistically significant difference between pN2a and pN2b, with 5-year OS rates of 63.3% and 56.3%, respectively (*p* = 0.083) [11], despite generally favorable outcomes in their cohort. Although absolute survival rates in these studies were higher than those in our PET/CT-based population, the magnitude of separation between N2a and N2b was modest, resembling the pattern seen in our analysis.

Several factors may account for these differences. The IASLC database includes a substantially larger number of patients, providing greater statistical power to detect survival differences between N2a and N2b. In contrast, smaller single- or multi-institutional cohorts, including ours and other validation studies, may not consistently demonstrate statistical significance even when similar trends are present. Moreover, pathological analyses in the IASLC study showed a higher hazard ratio than clinical staging subsets, suggesting that surgically confirmed nodal status may allow more accurate prognostic stratification than imaging-based assessment alone. Variability in surgical approach, the extent of lymph-node dissection, and the use of adjuvant or multimodality treatment, together with heterogeneity in definitive chemoradiotherapy and systemic therapy among clinically staged cohorts, may further influence both absolute survival rates and effect sizes across studies. Overall, our findings indicate that the separation between cN2a and cN2b appears less pronounced in an ^18^F-FDG PET/CT-based clinically staged population. Nevertheless, the direction of the observed effect is consistent with prior reports and continues to support the clinical relevance of lymph-node burden as incorporated in the ninth edition of the TNM classification.

One of the most notable changes in the 9th edition of TNM staging is the downstaging of T1N2a tumors from stage IIIA to stage IIB. This change is not a minor revision within stage III but a significant transition between stages with a completely different prognosis. The results of our study suggest that down-staging the staging of the T1N2a group from IIIA to IIB may not be appropriate. This is because the OS rate of patients in this group was significantly worse than that of patients classified as stage IIB from both staging systems (*p* = 0.029). Furthermore, the OS rates of these patients were similar to those of patients classified as stage IIIA in both TNM 8 and TNM 9 (*p* = 0.23). While TNM-9 generally provides improved prognostic stratification, our findings suggest caution when down-staging the T1N2a subgroup, because its survival outcomes appear closer to stage IIIA rather than to stage IIB. Notably, IASLC ninth-edition clinical staging analyses within the T1 subgroup reported 5-year OS of approximately 50% for cT1N2a and 43% for cT1N2b, suggesting that cT1N2a (stage IIB in TNM-9) has a more favorable prognosis than cT1N2b (stage IIIA in TNM-9) [5]. In addition, previous study reported 5-year survival rates of down-staged pT1N2a patients as high as 78.9%, with similar results to those of patients classified as stage IIB in both TNM 8 and TNM 9(71–77%) [12]; however, in our study, the 5-year survival rate of downstaged T1N2a patients was much lower (50.0%). This rate was closer to the stage IIIA group (46.0%) than the stage IIB group (61.0%).

In contrast, survival outcomes were more consistent with the new staging system in T1N1M0 patients whose stage was down-staged from IIB to IIA and in T2N2bM0 patients whose stage was up-staged from IIIA to IIIB. In particular, the survival rate of the patient group with stage IIB down-staged to IIA was higher than that of patients with stage IIB for TNM 8–9; however, this difference was not statistically significant (*p* = 0.19). This suggests that the change may be appropriate but needs to be validated in a larger group of patients. Furthermore, the survival rate of patients whose stage was upstaging from IIIA to IIIB was lower than that of those whose stage remained IIIA; However, this difference was also not statistically significant (*p* = 0.085). In addition, the survival rates of patients whose stage was up-staged from IIIA to IIIB were similar to those of IIIB patients whose stage had not changed since the beginning.

In our study, while the 5-year OS rate was 75.0% in patients who remained IIA according to both TNM 8 and TNM 9, this rate was higher (88.0%) in patients whose stage was down-staged from IIB to IIA. This finding was consistent with previous study; Nakao et al., who analyzed pathologically staged cohorts, reported a 5-year OS rate of 88.4% for patients down-staged from IIB to IIA and 80.2% for TNM 8–9 IIA [11]. This supports the idea that the new staging system accurately reflects the prognosis of this patient group. On the other hand, the most pronounced discrepancy between our findings and previous reports was observed in the patient group whose stage was up-staged from IIIA to IIIB. In the same pathologically staged cohort, the 5-year OS of patients up-staged from IIIA to IIIB was reported as 69.3%, whereas it was 39.3% among patients classified as stage IIIB in both TNM 8 and TNM 9 [11]. In contrast, in our PET/CT-based cohort, the 5-year OS of patients up-staged from stage IIIA to IIIB was 35.0%, compared with 39.0% in patients who remained stage IIIB, indicating an opposite trend. This discrepancy may be attributable to differences in patient selection, the use of pathological versus imaging-based nodal staging, or variability in treatment strategies between surgically staged and clinically staged populations.

Unlike previous studies, our study directly compared the prognosis of patient groups with stage transition according to the old and new staging systems. Thus, more precise information was obtained regarding the extent to which the new stages reflect the accurate prognosis of the patients. This method provided clinically important findings, especially regarding down-staging the T1N2a subgroup. Our data indicate that this patient group may not fully align with the newly assigned stage. Larger, multicenter studies are needed to confirm our findings and further improve lung cancer staging.

TNM 9 staging may also have important implications for treatment decisions for patient groups whose stages have changed. One of the most important changes in TNM 9, the down-staging of the T1N2a group from stage IIIA to stage IIB, may create significant differences in clinical practice [13–15]. Therefore, the down-staging of the T1N2a group from stage IIIA to IIB may put this patient group at risk of undertreatment.

Our study has some limitations. First, the study was conducted retrospectively using data from two different centers. In addition, multivariable survival analyses were not performed, as the primary objective of this study was to evaluate stage-based prognostic discrimination rather than independent treatment effects. Because treatment strategies were largely determined by stage, adjusting for treatment could have obscured the true prognostic contribution of stage classification. Nevertheless, potential confounding by treatment modality cannot be fully excluded. Another limitation is that invasive mediastinal staging methods were not used in some cN2 patients. Staging in these patients was performed using only ^18^F-FDG PET/CT, which may have affected the accuracy of nodal staging. Moreover, our relatively small sample size may have limited the statistical power. Although stage-migration analyses were performed in the overall cohort and histology-specific analyses were conducted for nodal subcategories, further stratification of stage-migration patterns according to histopathological subtype was not feasible because of limited patient numbers and reduced statistical power.

## Conclusion

The new TNM 9 classification divided the N2 stage into N2a and N2b subgroups, resulting in stage increases or decreases in some patient groups. The most significant change is the downstaging of the stage of the T1N2a group from III to II. In our study, the survival results of this patient group do not seem to be fully compatible with TNM 9; although other stage changes yielded results consistent with the new classification, they were not statistically significant. More comprehensive studies are needed to confirm these changes and their clinical consequences. Therefore, careful clinical judgment is warranted when managing T1N2a disease until further prospective validation becomes available.
